# Anorectal stenosis after treatment with tumor necrosis factor α antibodies: a case series

**DOI:** 10.1186/1752-1947-4-226

**Published:** 2010-07-26

**Authors:** Eoin Slattery, Denise Keegan, Diarmuid O'Donoghue

**Affiliations:** 1 Centre for Colorectal Disease, St. Vincent's University Hospital, Elm Park, Dublin 4, Ireland

## Abstract

**Introduction:**

We identified three patients who developed anorectal stenosis after successful treatment with anti-tumor necrosis factor α (anti-TNF-α) agents.

**Case presentation:**

Two patients, a 24-year-old Irish Caucasian man and a 64-year-old Irish Caucasian woman, developed symptoms attributable to anorectal stenosis four to six weeks after treatment. A further patient, a 25-year-old Irish Caucasian male, presented three years after treatment with anorectal stenosis, having been asymptomatic with his stenosis for the preceding three years. No patients had evidence of active inflammation at time of representation or had previous anal canal surgery.

**Conclusion:**

Anorectal stenosis in these patients appears to be independent of active inflammation. No other cause of new stenosis could be identified. We postulate that rapid clinical response to anti-TNF-α agents led to aberrant mucosal healing. This in turn led to anorectal stenosis. This is the first report of this complication in association with the use of biologic agents.

## Introduction

Tumor necrosis factor α antibodies (anti-TNF-α) have become widely used in the treatment of chronic inflammatory bowel disease (IBD). TNF-α is a proinflammatory cytokine that plays a central role in the pathogenesis of IBD. Currently, infliximab is licensed for use in moderate to severe Crohn's disease (CD) and ulcerative colitis (UC) [1,2]. Newer anti-TNF-α agents, including adalimumab and more recently certolizumab pegol, have been licensed for use in patients with CD.

Efficacy rates in excess of 40% to 60% have been reported for infliximab [3-6]. These potent drugs are tolerated well but are associated with potentially significant adverse effects. The most commonly reported adverse event associated with their use is an increased risk of infections [7,8]. Infusion reactions or anaphylaxis occur in a small proportion of patients (~5%) [6]. Most concerns about the use of these biologic agents relates to reports of hematologic and lymphoproliferative malignancies [9]. Concerns regarding the exacerbation of CD-associated small bowel strictures were raised when infliximab was first used.

We report a case-series of three patients who presented with anorectal stenosis after successful treatment with anti-TNF-α agents.

## Case presentation

### Case 1

Patient 1 is a 24-year-old Irish Caucasian man diagnosed with CD at the age of 18 years. Endoscopy at the time of diagnosis revealed active rectal and distal colonic inflammation and the presence of a draining perineal sinus. He was treated initially with a combination of steroids and subsequently maintained in remission with azathioprine. He has no other medical or surgical problems.

His disease became active two years later despite maintenance treatment with azathioprine. Repeat endoscopy confirmed active rectal and descending colonic inflammation; there was no evidence of active perineal disease. He was treated initially with an induction dose of infliximab (5 mg/kg at 0, 2 and 6 weeks) in the summer of 2005. He had a partial response and was continued again on azathioprine.

His disease flared again in 2007. At this time his laboratory, investigations suggested active disease (C-reactive protein [CRP] 53 mg/L, 0-4 mg/L is normal). This was confirmed by endoscopic evaluation, which revealed active left-sided colonic and rectal inflammation. He had no evidence of active perineal disease at this time. His Harvey-Bradshaw Index (HBI; score ≥7 indicates likely remission) was 9. Because of his partial response with infliximab, he was treated with adalimumab. He received 160 mg at induction and thereafter was maintained on 80 mg subcutaneously every other week. He made a rapid recovery, with resolution of his symptoms of diarrhea, pain and bleeding per rectum. His laboratory test results also improved (CRP <4) after treatment with adalimumab.

However, he presented one month after his commencement on adalimumab with 'diarrhea'again. His laboratory investigations revealed normal inflammatory markers (CRP <4), and his HBI had improved to 3. On further questioning, he admitted to feeling as well as he ever had. However, he was concerned about the recurrence of his 'diarrhea' and had started to become unaware of the passage of liquid stool. This was believed to be consistent with a diagnosis of 'overflowdiarrhea.' A rectal examination was attempted but was not possible because of anal stenosis.

He was admitted for endoscopic evaluation and was found to have a previously undocumented anal stenosis, with hard stool easily palpable above his stenosed anorectal canal. Endoscopy revealed a scarred but noninflamed colon. His anorectal stenosis was treated with a manual dilatation under anaesthesia, and he was subsequently discharged home. He required a further dilatation six weeks later. He has remained in clinical remission for three years maintained on adalimumab.

### Case 2

Patient 2 is a 64-year-old Irish Caucasian woman diagnosed with CD involving her entire colon (including active disease noted in her rectum) and orofacial granulomatosis. She had no evidence of perineal disease. She initially presented with diarrhea related to CD and was treated with corticosteroids. She was subsequently treated with azathioprine but was unable to tolerate it.

Her symptoms persisted and inflammatory markers deteriorated (erythrocyte sedimentation rate, 38 [reference range, 0-25 mm/h]; albumin, 27 [reference range, 35-50 g/L); hemoglobin, 9.4 (reference range, 13-17 g/dL); CRP, 9). Her HBI at this time was 7. She was subsequently started on infliximab (receiving 5 mg/kg at 0, 2 and 6 weeks). She again made a rapid recovery. Her symptoms and laboratory investigations all improved.

**Figure 1:**
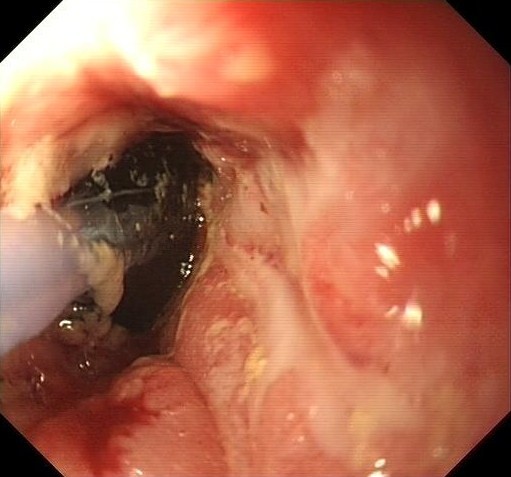
**Balloon dilatation of anorectal stenosis**. This endoscopic picture shows the stenosis seen in patient 2. A balloon has been advanced through the stricture and has been inflated in the anorectal canal to dilate the patient's stenosis.

However, she presented one month after starting on infliximab with a new problem of constipation. Again, on clinical examination, she was found to have a stenosed anal canal. Her laboratory parameters had all normalized, as had her HBI to 1. She was admitted for endoscopic evaluation and was found to have a stenosed anorectal canal with a noninflamed colon (Figure 1). Her stricture was treated using balloon dilatation, and she was discharged home with resolution of her symptoms. She was maintained on infliximab (at a dosage of 5 mg/kg every 8 weeks). Unfortunately, she has required a further five dilatations of her anorectal canal, with temporary interval improvement. She continued to have problems and eventually required a diversion colostomy.

### Case 3

Patient 3 is a 25-year-old Irish Caucasian man diagnosed with CD colitis affecting the descending colon and rectum. He presented with active disease three years previously. Endoscopic evaluation revealed active rectal and colonic inflammation; his perineum appeared normal. His laboratory investigations confirmed active inflammation with a CRP of 38. His HBI was 10 at the time of presentation. He was treated initially with infliximab (5 mg/kg at 0, 2 and 6 weeks) after failed corticosteroid treatment. His disease responded rapidly. After he was in remission (after three doses of infliximab), he was switched to azathioprine and maintained on it. At a follow-up colonoscopy eight weeks after starting infliximab, we noted a stenosed anorectal canal with a quiescent colitis proximal to this. In the intervening three years, the patient has remained well. He recently represented with severe abdominal pain and constipation. His laboratory investigations were all normal (CRP <4), and his HBI was calculated to be 2. He was found to have an anorectal stenosis with hard stool proximal to this. He was treated with mechanical bowel preparation, and a follow up colonoscopy revealed a normal colon above a stenosed anorectal canal. He was subsequently treated with a balloon dilatation of his stricture. His CD remains in remission.

## Discussion

Benign anal stenosis is a rare complication of previous anal surgery and is occasionally seen in patients with active CD. Benign anal stenosis in the absence of previous surgery after successful treatment with anti-TNF-α agents has not previously been reported.

Theoretical concerns have been raised regarding the occurrence of small bowel stricturing as a consequence of rapid mucosal healing. There have been reports of increased episodes of stricturing in some studies [10,11]. However, multivariate analysis has demonstrated that infliximab use is not associated with increased small bowel stricture development when corrected for confounders such as disease duration, disease severity and so on [11]. More recent studies have actually demonstrated a potential benefit to patients with small bowel strictures [12,13].

The precise role of TNF-α in healing is not well established. It has been shown that persistently elevated TNF-α levels in patients with chronic skin ulcers may impair healing and that treatment with infliximab may reverse this process rapidly [14]. We postulate that rapid clinical response to these potent biologic agents led to rapid but aberrant mucosal healing at and around the dentate line between anus and rectum (as opposed to isolated bowel strictures). This in turn led to development of anorectal stenosis in these patients. None of these patients had prior anal canal surgery or had evidence of active perineal CD (eg, abscess, fistula) at the time of representation (either endoscopically or biochemically). All had colonic and rectal inflammation before starting anti-TNF-α agents. No other precipitant cause for anal canal stenosis could be determined in these cases.

Two of our patients presented with symptoms four to six weeks after administration of these biologic agents. The third patient presented much later, but he was found to have a degree of anal stenosis at follow-up endoscopy shortly after administration of infliximab. At this stage, he was asymptomatic and thus required no intervention. However, this likely made him prone to future problems with anorectal stenosis and explains his later presentation.

## Conclusion

This is the first report of anorectal stenosis in association with CD after successful treatment with biologic agents and in the absence of persisting active inflammation. Further research on the role of TNF-α and its blocking agents in wound healing is warranted.

## Consent

Written informed consent was obtained from the patients for publication of this case series and accompanying images. A copy of the written consent is available for review by the Editor-in-Chief of this journal.

## Competing interests

The authors declare that they have no competing interests.

## Authors' contributions

ES and DK analyzed and interpreted the patient data, ES wrote the manuscript. DO was a major contributor in writing the manuscript. All authors read and approved the final manuscript.
